# Physiological Considerations in Transfusion of Blood

**Published:** 1876-05

**Authors:** C. George

**Affiliations:** Ann Arbor, Mich.


BUFFALO

### Medical and Surgical Journal.

VOL XV. MAY, 1876. No. 10.

### Original Communications.

———:o:———

ART. I.—*Physiological Considerations in Transfusion of Blood.* By C. GEORGE, M. D., Ann Arbor, Mich.

Transfusion, apparently a new remedy, has already passed its second centennial. Hailed with great enthusiasm when first introduced, it soon shared the fate of all new remedies whose praise is solely due to empirical knowledge derived from their history in a few cases. It so came into disfavor that it was forbidden in practice by law, and thus it passed for a time into utter oblivion to be resusictated by modern physicians. Now at the dawn of a new career it is again blessed with the empirical praise of physicians and surgeons, while the voice of the physiologist is almost unheeded. Although the knowledge of the therapeutical indications of a remedy derived from empirical practice is not to be ignored, yet it is equally true that inductive knowledge of such indications derived from experimental investigations should at least be regarded with equal candor.

The questions at issue pertain to the therapeutical indications, and the kind of blood to be used—here we find the voice of empiricism in favor of using it almost promiscuously, and of employploying any kind of blood, alkaline solutions, milk, etc., while the physiologist demands human blood and that for certain specific indications only.

I propose to render a resumé of the views of the physiologists of the day in regard to these vital questions.

While it is generally conceded that the blood takes up oxygen as oxyhæmaglobin in its passage through the lungs, and that it is the carrier of nutritive elements derived in part directly from the stomach and intestines and indirectly through the thoracic duct, that it again in turn relieves the tissues from effete materials by conveying these substances to the excretory glands ; it is still held by some physicians that the blood is more than a ready vehicle for oxygen and nutritive elements in their passage to the tissues and for effete material in its passage to the emumctories, that it serves also as a direct nourishment through the albuminoids contained in the corpuscles and plasma in case that the requisite supply is not furnished by the proper organs. This view was derived from an observation of Bidder and Schmidt, who observed that the diminution of the blood, in quantity and quality, of a starved animal was greater than that of the body and at once concluded from this single observation that the blood served as nourishment. This opinion has exercised great influence on the therapeutical application of transfusion in the past and has not yet lost all sway. In conformity with this view, patients lying in the last stages of consumption have been, and are still treated with transfusion in the vain hope of enlivening the system with healthy blood. It is further practiced in various forms of chronic anæmia caused by incurable disease of the nutritive organs, and anæmia caused by fevers, etc.

Recent investigations by Panum have demonstrated the fallacy of this doctrine beyond the limits of reasonable doubt. (*Virchow's Archiv.*) His experiments are fully corroborated by those of Ponfick and Mittler. All recent investigators have come to the conclusion that in inanition the red corpuscles of the blood do not diminish more in proportion than the body itself, that transfusion will only increase the relative diminution of the weight of the body on account of the increased amount of oxygen required for the transfused blood; that the amount of fibrine is not diminished, that it is soon reproduced to the original amount if depletion precedes transfusion of defibrinated blood in independent of the presence of inflammatory processes; and finally that the albuminoids diminish somewhat in amount, yet this diminution is not constantly observed ; and that the relative amount of diminution is so slight, when it does take place, as to prove efficiently that the albuminoids are not consumed by the tissues.

The physiological action of albuminoids, however, is as yet but very imperfectly understood. It is probable that they are the carriers of water. There are at least three different kinds of albuminoids, viz.: fibrine which separates readily on coagulation, serum casein, which is obtained after removing the fibrine by diluting and neutralizing the serum, and lastly serum albumen, which remains after removing the two former varieties, and is freed by boiling, alcohol, &c. It is thought that the serum albumen is the vehicle for the circulating water and the salts in solution in the plasma, and that the serum casein, which is probably produced by the corpuscles, serves as immediate nourishment i. e. as material for reproduction. Fibrine is probably produced outside the vessels as a secondary product during cell formation and serves probably as material for certain secretions. This view is fully sustained by the fact that in all inflammatory processes the amount of fibrine is not diminished, but augmented as long as the inflammation is accompanied by increased cell production. The fact that the relative amount of fibrine is not diminished during inanition unaccompanied by inflammatory action, is still further support for the above theory.

Next in order we shall consider the part performed by the individual constituents of the blood in the respiratory process. The serum and plasma of the blood absorb as much oxygen as water, under equal pressure of temperature, i. e., 2–3 per cent. by volume. Hæmaglobine, an essential constituent of the red corpuscle unites chemically with oxygen, forming oxyhæmaglobine. The amount of oxygen which the blood is thus capable of absorbing depends chiefly on the number of red corpnscles, respectively on the amount of hæmaglobine which it contains. This process does not depend on either pressure or temperature. Oxyhæmaglobine is readily decomposed by carbonic acid gas on account of the greater affinity of this substance for the oxygen than the hæmaglobine. The tissues receive their requisite supply of oxygen during the passage of the blood through the capillaries. Arterial blood is normally saturated with oxygen and this is still the case when the respired air contains only one-half the usual amount of oxygen, and again hæmaglobine does not combine with more oxygen, even if pure oxygen is respired, nor is the amount of oxygen consumed in twenty-four hours increased by inhaling pure oxygen. Respiration first becomes impaired when the oxygen in the respired air is reduced to one-third that of the usual quantity and suffocation is readily induced by air containing only from 3–5 per cent. by volume. Freed hæmaglobine takes no share in the respiratory process, it is a foreign substance and is carried off by the kidneys. Venous blood contains about 5 per cent. by volume less of oxygen than arterial blood, which consequently represents the amount of oxygen received by the tissues. Hence venous blood contains a comparatively large amount of dispensable oxygen and it first looses all traces of it when death has been caused by complete occlusion of the respiratory passages. In such cases we scarcely find a trace of oxygen in the air of the respiratory passages.

Carbonic acid is carried off by the water and salts of the plasma and particularly the alkaline carbonates and phosphates. Arterial blood is not free from carbonic acid, but contains usually from 26-30 per cent. by volume, whilst venous blood contains from 2–6 vol. more, this then is the amount taken from the tissues. The amount of carbonic acid found in the blood when death has been caused by suffocation, never exceeds 53 per cent. by volume. Venous blood is never saturated with carbonic acid, it can take under the usual barometric pressure and medium temperature from 150–180 per cent. by volume whilst water under the same conditions takes only about 100 per cent. by volume. The amount of carbonic acid, however, is increased when a mixture of carbonic acid and oxygen is inhaled, as is the case when an animal is subjected to such a mixture in a hermetically sealed reservoir. Death when thus produced differs essentially from that caused by the occlusion of the respiratory tract, this is carbonic acid poisoning, which is not accompanied by convulsions.

The amount of carbonic acid exhaled by the lungs is free, i. e., simply absorbed by the water ; another part which is held chemically as sesqui and bicarbonates, is liberated by boiling in air-tight covers, and the residue is combined as carbonates. Oxyhæmaglobine acts as an acid on the carbonates, liberating a greater part of $CO_2$ held loosely as bicarbonates, and also part of that held by the carbonates.

In defibrinated blood the red corpuscles have not lost the property of combining with oxygen, while the serum and its salts still absorb carbonic acid, and if left standing it yields oxygen and absorbs carbonic acid precisely like the original fluid. Defibrinated venous blood is for all practical purposes identical with defibrinated arterial blood ; should carbonic acid predominate it can easily be expelled by stirring the fluid rapidly in contact with pure air, when it will at once assume the bright scarlet color of fresh arterial blood. Defibrinated blood can be preserved for at least 24 hours in an air-tight vessel secured in ice. Blood thus preserved has simply to be raised to the normal temperature and saturated with oxygen before being employed.

There is a marked difference in the amount of oxygen which defibrinated blood is capable of absorbing, this depends entirely on number of corpuscles removed by the defibrinating process. That defibrinated blood is capable of performing all the functions of the vital fluid has been amply demonstrated by the experiments of Panum and Ponfick. These investigators subjected dogs to repeated depletions and transfusions of similar defibrinated blood until there could scarcely have been left more than a trace of the original blood, the dogs appeared active and lively during these experiments and when finally killed the amount of fibrine was only slightly in arrear of the original amount. These experiments were not accompanied by inflammatory action.

To sum up—the red corpuscles by virtue of their hæmaglobine take up oxygen and convey it to the tissues, the water of the plasma is the vehicle for the nutritive elements and salts and conveys the carbonic acid and other products of waste to the proper emunctories. It seems also that it is the function of the albuminoids contained in the plasma to absorb and retain water, being thus the chief vehicle for the water received during alimentation and also for that excreted by the kidneys, skin, etc.

Grave complications, so frequently observed after the introduction of large quantities of blood have heretofore been attributed to the sudden increase of arterial pressure. This doctrine has had a grave influence on the therapeutics of transfusion,—it has induced practitioners to deplete before transfusing.

M. Herman has demonstrated that spring or distilled water can not be used for transfusion because it dissolves a proportionate amount of red corpuscles. Ponfick did not observe grave symptoms following alkaline solutions even in large quantities when the experiment was conducted slowly. By using an artificial serum (1 p. ct. Na. Cl. and albumen of egg.) in large quantities he observed slight depression, which, however, subsided shortly after relieving the animals. The urine was always peculiarly affected, the kidneys did not manifest any symptoms indicative of augmented arterial pressure, the amount of urine was not perceptibly increased; its sp. gr. however was greatly reduced, had an alkaline reaction and contained large quantities of albumen of a foreign variety, deficient in urea. The alkaline reaction subsides with the disappearance of the albumen, simultaneously with these changes the urea increases in amount and the sp. gr. rises to its original height. The increased pressure of the vascular system however could not have been reduced by the exhalations of the skin, or discharges of the bowels, for both were very slight. In these experiments Ponfick did not observe the bloody exudations of former authors; they have to be attributed to other causes than augmentation of pressure.

Ponfick obtained very different results with natural serum; urine remains acid, sp. g. not affected, amount passed almost unaltered, no trace of albumen. These results are corroborated by the experments of Stockvis, Leman and Worm-Mueller.

Worm-Mueller in his experiments observed a considerable increase of pressure at the carotids immediately after the operation, subsiding, however, soon afterwards. He sums up his result as follows: "It is highly probable that the vascular system can accommodate itself within certain limits to larger or smaller quantities of blood without giving rise to material change in pressure or abnormal dilitation of the walls of the vessels, or any serious symptoms whatever." He attributes this function of adaptation of the vascular system to the influence of the vaso motor nervous system.

The danger of inducing an acute plethora by transfusion has thus been decidedly overestimated, the symptoms solely due to it are far too insignificant to warrant the heroic procedure of previous depletion; and these very symptoms may follow a preceding depletion if the transfusion is conducted too rapidly. Depletion however might be practiced with benefit if we could demonstrate with certainty that the corpuscles are undergoing disintegration, as in carbonic acid gas poisoning, in such cases the corpuscles are worse than useless.

Transfusions with similar defibrinated blood are not followed by any abnormal symptoms, contrarily when preceded by depletion they produce augmented animation. The amount of urine is not increased, color, spec. grav. and reaction remain normal, no trace of albumen detected. Direct transfusions with similar blood do not materially differ from those of defibrinated blood if no coagala are injected.

Transfusions with different kinds of blood, whether defibrinated or not, have had very different results from the above in the hands of all modern investigators.

Dyspnœa ensues rapidly as the operation progresses, manifested at first by increased frequency of respiration, it is soon accompanied by convulsive movements of the diaphragm and later by general convulsions. These symptoms are accompanied by frequent stools and severe vomiting which in time give place to tenesmus and intense nausea. There is complete suppression of urine and hæmaglobinurea is quickly announced by the appearance of a few bright red drops of dissolved hæmaglobine and cylinders, scales, etc., (catheter is always used). Depression follows rapidly, the respiratory movements become superficial and less freqeunt and all the muscles of the body relaxed. The released animals lie in a state of apathy and gloomy silence, broken from time to time by faint dismal groans until death terminates the scene. In section the mucous membrane of the stomach duodenum and of the entire ileum is found of dark red color, greatly swollen and covered by a thick reddish mucus. The villi are swollen and indistinct, no discrete bloody effusions in their tissue. The same condition continues in increased intensity to the colon and reaches its height in the sigmoid flexure. Numerous hemorrhages in the mucous membrane. Rectum is similarly affected. The kidneys however contain the chief lesions caused by dissimilar blood, when enough is injected to produce any pathological condition whatever. They are greatly swollen, but not always infiltrated with blood; on the contrary the texture, particularly towards the periphery, appears frequently very pale of dirty greyish brown color. The capsule peels off readily and numerous sharply bordered spots and stripes of a reddish or coffee brown color are seen imbedded in the smooth distended surface of the peculiar brownish ground-texture of the organ. The appearance as well as the distribution of these spots and stripes reminds one of the multiple spots of hæmorrhagic nephritis. On section they appear in great numbers at the periphery, but less sharply contoured from the surrounding parenchyma, which is greatly swollen, very pale and of uniform color. Ferrein's pyramids render the inner half of a coarsely striated appearance. The Malpighian bodies appear shrunken on account of the slight amount of blood they contain. The papillæ (Markkegel) are very large and bloody toward the periphery, and the brown and red stripes raidiating towards them alternate regularly. In severe cases the brownish color is so predominant that the reddish lines can scarcely be recognized. A dark brown or light colored fluid exudes from the papillæ on pressure in which small bodies are distinctly seen suspended. A similar fluid varying in amount is found in the pelvis of the kidneys and bladder. Fatal cases are characterized by absolute emptiness of the urinary reservoirs. The adipose tissue of the hilus is frequently remarkably œdematous.

Microscopical investigation has shown that all the brown spots and stripes in the cortical substance and the radiating lines in the pyramids are caused by solid plugs in the tubuli uriniferi. The color of these cylinders corresponds at first, exactly with the red corpuscles; in the latter stages it assumes a darker and brownish tint. This does not depend on the presence of colored cells in the tubuli but on an imbibition of either a hyaline or corpuscular substance and hæmaglobine.

Bloody transuadations in the stomach and intestinal tract have indeed also been observed to follow transfusions with similar blood, yet these have to be attributed to very different factors, for they do not always appear nor are they permanent in character. In direct transfusion with similar blood we must take into consideration the great danger of introducing small coagula, or of transfusing the blood to rapidly ; either of these factors is capable of producing all the above symptoms,—in transfusing defibrinated blood he must bear in mind that it is an easy matter to leave small particles of coagulated fibrine behind or of contaminating the blood otherwise. There is also great danger of introducing air with the frequent introduction of the syringe. Transfusions with similar blood have been performed without giving rise to any symptoms otherwise than those indicative of heightened animation. Transfusions with dissimilar blood whether defibrinated or not have always given rise to grave symptoms and the gravity depended on the amount introduced; and there is one grand factor which distinguishes the whole train of symptoms which may follow transfusions of similar blood from those which constantly follow transfusions of dissimilar blood, i. e. in the former the urine does not present any changes indicative of either kidney disease or destruction of the red corpuscles, whereas in the latter the urine always shows the peculiar action of dissimilar blood, and finally death may be caused by dissimilar blood without giving rise to those affections of the stomach and intestines which are the chief lesions observed after transfusions with similar blood.

Ponfick has demonstrated by chemical, microscopic and spectroscopic examination that the reddish color of the urine does not depend on the presence of red corpuscles but on loose hæmaglobine. Whenever he found red corpuscles he readily accounts for them by the very frequent catheterism to which the animals were subjected. The urine also contains brownish cylinders, the tubuli uriniferi. During the presence of the hæmaglobine urine had an alkaline reaction and of low sp. gr. Thus we see an unmistakable antagonism between the exudation of the hæmaglobine and the normal action of the kidneys, the normal constituent of the urine being conspicuously absent during the presence of the hæmaglobine, and first reappearing a few hours after the last traces of hæmaglobine have disappeared. The quantity of hæmaglobine, the number of cylinders and the degree of reduction of sp. grav. stand in direct relation to the amount of dissimilar blood used for transfusion.

It is regarded by physiologists as a certainty that the hæmaglobine comes from the corpuscles of the foreign blood, that all dissimilar corpuscles undergo disintegration, and that they dare not take part in the respiratory process. When transfusions of such small quantities of dissimilar blood as do not produce hæmaglobinurea are repeated at comparatively short intervals the combined effect will appear as soon as such a quantity has been transfused as would produce hæmaglobinurea if injected at once.

The appearance of hæmaglobinurea is the beginning of the end —if the quantity of hæmaglobin excreted by the kidneys is so slight that it does not materially disturb the function of these organs then the victim will survive, but should the quantity be so great as to block up and destroy great number of tubules at once then death must inevitably terminate the scene.

Prevost and Dumas state that all dissimilar blood has a poisonous effect. Ponfick, Panum, Mittler and Landois have corroborated this statement so far in demonstrating experimentally that the blood of the following animals, viz: lamb, hog, calf, cat, also that of hen, rabbit and duck, etc., had a poisonous effect on the dog. Ponfick also proved that human blood had the same effect on the dog, so man cannot be regarded an exception to this physiological law and patients have actually expired immediately after lamb blood transfusions who, in all human probability, would have lived.*

In conclusion, it seems hardly necessary to state that transfusion is, as far as known at present, only indicated in cases of severe and fatal hemorrhage, that human blood alone will serve the purpose and that defibrinated blood has the advantage in being capable of performing all the functions of the vital fluid without endangering the system through coagula so readily produced in transfusions with original blood.

---

* See Denis' Lamb blood transfusion.

